# Evaluation of the efficacy and safety of immunotherapy in sarcoma: a two-center study

**DOI:** 10.3389/fimmu.2024.1292325

**Published:** 2024-03-22

**Authors:** Zhichao Liao, Jianjin Teng, Tao Li, Haotian Liu, Ting Li, Chao Zhang, Ruwei Xing, Sheng Teng, Yun Yang, Jun Zhao, Wanyi Xiao, Gengpu Zhang, Mulin Jun Li, Weitao Yao, Jilong Yang

**Affiliations:** ^1^ Department of Bone and Soft Tissue Tumor, Tianjin Medical University Cancer Institute and Hospital, Tianjin, China; ^2^ National Clinical Research Center for Cancer, Key Laboratory of Cancer Prevention and Therapy, Tianjin’s Clinical Research Center for Cancer, Tianjin Medical University Cancer Institute and Hospital, Tianjin, China; ^3^ Department of Bioinformatics, School of Basic Medical Sciences, Tianjin Medical University, Tianjin, China; ^4^ Department of Bone and Soft-Tissue Tumor, Institute of Cancer and Basic Medicine, Chinese Academy of Sciences, Cancer Hospital of the University of Chinese Academy of Sciences, Zhejiang Cancer Hospital, Hangzhou, China; ^5^ Department of Bone and Soft Tissue Cancer, The Affiliated Cancer Hospital of Zhengzhou University & Henan Cancer Hospital, Zhengzhou, China

**Keywords:** sarcoma, immunotherapy, efficacy, adverse event, two-center

## Abstract

**Background:**

Sarcoma is a highly heterogeneous malignancy with a poor prognosis. Although chemotherapy and targeted therapy have improved the prognosis to some extent, the efficacy remains unsatisfactory in some patients. The efficacy and safety of immunotherapy in sarcoma need further evaluation.

**Methods:**

We conducted a two-center study of sarcoma patients receiving PD-1 immunotherapy at Tianjin Medical University Cancer Institute and Hospital and Henan Provincial Cancer Hospital. The treatment regimens included PD-1 inhibitor monotherapy and combination therapy based on PD-1 inhibitors. The observed primary endpoints were median progression-free survival (mPFS) and median overall survival (mOS). Survival curves were compared using the Kaplan−Meier method.

**Results:**

A total of 43 patients were included from the two centers. The median follow-up time for all patients was 13 months (range, 1-48 months). In the group of 37 patients with advanced or unresectable sarcoma, the mPFS was 6 months (95%CI: 5-12 months), and the mOS was 16 months (95%CI: 10-28 months). The ORR was 10.8% (4/37), and the DCR was 18.9% (7/37). Subgroup analysis showed no significant differences in mPFS (p=0.11) and mOS (p=0.88) between patients with PD-L1 negative/positive expression. There were also no significant differences in mPFS (p=0.13) or mOS (p=0.72) between PD-1 inhibitor monotherapy and combination therapy. Additionally, there were no significant differences in mPFS (p=0.52) or mOS (p=0.49) between osteogenic sarcoma and soft tissue sarcoma. Furthermore, the results showed no significant differences in mPFS (p=0.66) or mOS (p=0.96) between PD-1 inhibitors combined with targeted therapy and PD-1 inhibitors combined with AI chemotherapy. Among the 6 patients receiving adjuvant therapy after surgery, the mPFS was 15 months (95%CI: 6-NA months), and the mOS was not reached. In terms of safety, most adverse events were mild (grade 1-2) and manageable. The most severe grade 4 adverse events were bone marrow suppression, which occurred in 4 patients but resolved after treatment. There was also one case of a grade 4 adverse event related to hypertension.

**Conclusion:**

Immunotherapy is an effective treatment modality for sarcoma with manageable safety. Further inclusion of more patients or prospective clinical trials is needed to validate these findings.

## Background

Sarcomas compose a large and unique group of rare malignant tumors with high heterogeneity; there are more than 100 subtypes, which can be divided into osteogenic sarcoma and soft tissue sarcoma (1, 2). The prognosis of patients with sarcoma is poor, and the 5-year overall survival rate is approximately 60%. Surgery and comprehensive treatment based on AI chemotherapy have greatly improved the prognosis of patients with sarcoma. However, there are still a considerable number of sarcoma subtypes that are not sensitive to chemotherapy, and patients with advanced metastatic disease, especially those with lung metastasis, have limited treatment options (3). In recent years, the application of anti-angiogenesis targeted drugs, such as apatinib and anlotinib, has further improved the survival of patients with sarcoma to some extent, but patients often develop drug resistance after a period (4–7). Therefore, in addition to conventional surgery, chemotherapy and targeted therapy, new therapeutic methods are still needed to fight this malignant tumor.

Immunotherapies – including immune checkpoint inhibitors, CAR T cells, and cancer vaccines – are an effective option for the control of advanced malignant tumors. Among them, immune checkpoint inhibitors, mainly PD-1/PD-L1 inhibitors, have been widely used (1). With the development of a series of clinical trials, such as SARC-028, immunotherapy has officially been introduced as a treatment option for sarcoma. However, as an immune “cold” tumor, the efficacy of PD-1/PD-L1 monotherapy in sarcoma is poor, and the efficacy of different pathological subtypes is different (8, 9). For example, in the SARC-028 trial, the response rate of soft tissue sarcomas was 18% (7/40), of which undifferentiated pleomorphic sarcoma had the highest response rate of 40% (4/10), followed by liposarcoma (20%, 2/10); none of the 10 leiomyosarcoma patients had a response (0%), and the response rate of osteogenic sarcoma was 5% (2/40) (10). In other clinical trials, the subtype with a better response rate includes alveolar soft part sarcoma (11, 12). However, in the field of sarcoma, there are still some problems that need to be solved, such as whether PD-1/PD-L1 inhibitors combined with other treatments can improve the efficacy compared with PD-1/PD-L1 inhibitor monotherapy, whether there is a difference in the efficacy of combined chemotherapy or combined targeted therapy, and whether adverse events are related to prognosis.

In this study, we retrospectively collected data from patients treated with PD-1 immune checkpoint inhibitors at two centers, evaluated their overall efficacy and safety, and analyzed them separately according to their PD-L1 expression, the use of monotherapy versus combination therapy, and whether adverse events occurred in order to better guide clinical practice.

## Materials and methods

### Patients and treatment

The study was a two-center study that included 36 patients with sarcoma at Tianjin Medical University Cancer Hospital from June, 2016 to May, 2022 and seven patients with sarcoma at Henan Province Cancer Hospital from March, 2019 to November, 2021. Based on medical records, we retrospectively collected information on all patients, with all pathology confirmed by two pathologists.

In this study, the immunotherapy drugs used were PD-1 inhibitors, including nivolumab, pembrolizumab, camrelizumab and toripalimab. PD-L1 inhibitors and CTLA-4 inhibitors were not included. Anti-angiogenesis targeted therapy includes apatinib, anlotinib and pazopanib; and chemotherapy regimens are AI (ifosfamide combined with adriamycin) and albumin-bound paclitaxel combined with gemcitabine. All patients received at least 2 cycles of PD-1 immunotherapy. The combination therapy regimens include immunotherapy combined with chemotherapy, and immunotherapy combined with targeted therapy, such as toripalimab combined with AI chemotherapy, and camrelizumab combined with apatinib, etc.

The treatment plan for patients was formulated based on a comprehensive consideration of the pathological subtype and the efficacy of previous treatments. For sarcoma subtypes that are insensitive to chemotherapy, such as alveolar soft part sarcoma, immunotherapy was used. For other sarcomas that are highly or moderately sensitive to chemotherapy, such as rhabdomyosarcoma and osteosarcoma, chemotherapy remains the first-line treatment. Immunotherapy is considered only after chemotherapy failure.

### Efficacy evaluation

Efficacy evaluation mainly includes short-term efficacy evaluation and long-term efficacy evaluation. Short-term efficacy was mainly evaluated at 12 weeks, including complete response (CR), partial response (PR), stable disease (SD), progressive disease (PD), objective response rate (ORR), and disease control rate (DCR). Long-term efficacy refers to the efficacy at the end of follow-up, and indicators included median progression-free survival (mPFS) and median overall survival (mOS) in addition to the above indicators. ORR was calculated as follows: (CR + PR)/total number of cases ×100%. DCR was calculated as follows: (CR + PR + SD)/total number of cases ×100%. PFS was defined as the time from the start of treatment to disease progression. OS was defined as the time from the start of treatment to death from any cause. mPFS and mOS were the primary endpoints of this study.

We assess the target lesions based on imaging examinations and RECIST 1.1 criteria, such as progressive disease is defined as an increase of 20% or more in the sum of the diameters of target lesions, or the emergence of new lesions. Imaging assessments were conducted every 8 weeks, evaluated by experienced radiologists and clinical physicians. When patients experienced adverse events, clinical physicians assessed their tolerance to immunotherapy. If tolerable, patients could continue immunotherapy according to the original plan with symptomatic treatment. If intolerable, immunotherapy was terminated.

### Safety and toxicity assessment

All patients were evaluated for safety and toxicity and graded according to Common Terminology Criteria for Adverse Events (CTCAE, version 5.0).

### The evaluation criteria for PD-L1 detection

In this study, immunohistochemistry (IHC) was used to evaluate the expression of PD-L1. The Tumor Cell Proportion Score (TPS) was used, which is the percentage of stained positive tumor cells among all tumor cells. The formula for calculating TPS is TPS = (Number of PD-L1 membrane-stained positive tumor cells/Total number of tumor cells) * 100%. A TPS of less than 1% is defined as no PD-L1 expression, a TPS range of 1-49% is defined as PD-L1 expression, and a TPS of 50% or higher is defined as high PD-L1 expression.

### Statistical analysis

All data were analyzed using SPSS 22.0 and R4.2.2. The Kaplan−Meier method was used to compare the differences between survival curves, and p values <0.05 were considered statistically significant.

## Results

### Patient demographics

A total of 43 patients were included in the study, including 36 patients from Tianjin Medical University Cancer Hospital and 7 patients from Henan Province Cancer Hospital (**Table 1**). There were 25 males and 18 females, with a median age of 49 years (range, 14-78 years). The median follow-up time for all patients was 13 months (range, 1-48 months). The immunotherapy regimen included PD-1 monotherapy, PD-1 combined with anti-angiogenic targeted therapy, PD-1 combined with chemotherapy, PD-1 inhibitors + targeted therapy + chemotherapy, and PD-1 inhibitors + targeted therapy + radiotherapy. There were 15 pathological subtypes, including osteogenic sarcoma and soft tissue sarcoma. The most common were undifferentiated pleomorphic sarcoma (7 cases), liposarcoma (5 cases), and osteosarcoma (5 cases).

**Table 1:** Patient Characteristics.

Characteristics	Case number (%)
Age	
Range	14-78
Median	49
Gender	
Male	25(58.1%)
Female	18(41.9%)
Pathology	
Malignant solitary fibrous tumor	1(2.3%)
Malignant peripheral nerve sheath tumor	2(4.7%)
Osteosarcoma	5(11.6%)
Rhabdomyosarcoma	3(7%)
Synovial sarcoma	3(7%)
Leiomyosarcoma	4(9.3%)
Chondrosarcoma	2(4.7%)
Epithelioid sarcoma	3(7%)
Clear cell sarcoma	2(4.7%)
Undifferentiated pleomorphic sarcoma	7(16.3%)
Undifferentiated sarcoma	1(2.3%)
Fibrosarcoma	1(2.3%)
Alveolar soft part sarcoma	3(7%)
Angiosarcoma	1(2.3%)
Liposarcoma	5(11.6%)
Therapeutic regimen	
Advanced or unresectable	37(86%)
PD-1 inhibitors monotherapy	4
PD-1 inhibitors + targeted therapy	24
PD-1 inhibitors + chemotherapy	6
PD-1 inhibitors + targeted therapy + chemotherapy	2
PD-1 inhibitors + targeted therapy + radiotherapy	1
Adjuvant therapy	6(14%)
PD-L1 expression	
Positive	3(7%)
Negative	10(23%)
Unknown	30(70%)

### Efficacy of 37 advanced or unresectable patients

Considering that 6 of the 43 patients received postoperative adjuvant therapy and were in a tumor-free state, we analyzed the efficacy of the remaining 37 patients who were at an advanced stage or unresectable, and we analyzed the 6 patients who received postoperative adjuvant therapy separately. The efficacy of 37 patients with advanced or unresectable tumors is shown in the **Supplementary Table 1**.

**Figure 1:**
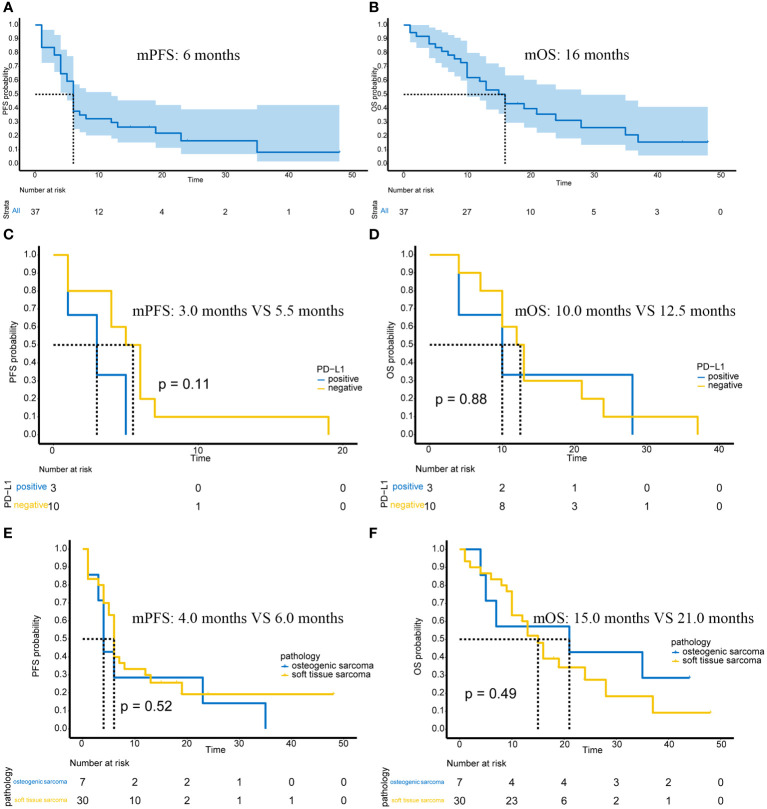
Efficacy of immunotherapy in advanced or unresectable patients. **(A)** The mPFS of all patients; **(B)** The mOS of all patients; **(C)** PFS in PD-L1-positive/negative patients; **(D)** OS in PD-L1-positive/negative patients; **(E)** PFS in patients with osteogenic sarcoma and soft tissue sarcoma; **(F)** OS in patients with osteogenic sarcoma and soft tissue sarcoma.

In 37 advanced or unresectable patients, the mPFS was 6 months (95%CI: 5-12 months) (**Figure 1A**). The mOS was 16 months (95%CI: 12-28 months) (**Figure 1B**). At 12 weeks, 1 patient achieved complete response (CR), 5 patients achieved partial response (PR), 21 patients had stable disease (SD), and 10 patients had progressive disease (PD). The overall response rate (ORR) was 16.2% (6/37), and the disease control rate (DCR) was 73%. By the end of the follow-up, there were 3 patients with CR, 1 patient with PR, 3 patients with SD, and 30 patients with PD. The ORR was 10.8% (4/37), and the DCR was 18.9% (7/37).

Of the 37 patients, 13 underwent PD-L1 immunohistochemical testing, of which 3 were positive and 10 were negative. The high and low expression of PD-L1 is shown in **Supplementary Figure 1**. Possibly because the number of cases was too small, there was no significant difference in PFS (p=0.11) (**Figure 1C**) or OS (p=0.88) (**Figure 1D**) between PD-L1 positive and negative patients.

In terms of pathological subtypes, there were 7 cases of osteogenic sarcoma, including 5 cases of osteosarcoma and 2 cases of chondrosarcoma. There were also 30 cases of soft tissue sarcoma. There were no significant differences in PFS (p=0.52) (**Figure 1E**) or OS (p=0.49) (**Figure 1F**) between osteogenic sarcoma and soft tissue sarcoma patients.

**Figure 2:**
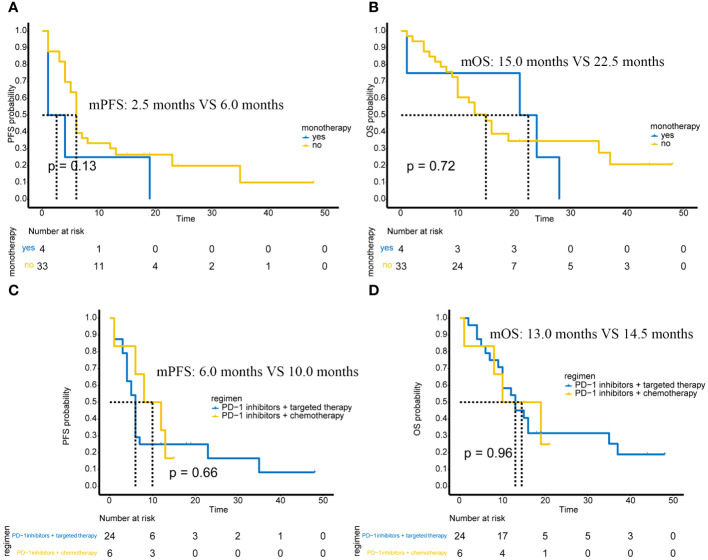
Efficacy in different subgroups of advanced or unresectable patients. **(A)** PFS in PD-1 inhibitor monotherapy and PD-1 inhibitor-based combination therapy; **(B)** OS in PD-1 inhibitor monotherapy and PD-1 inhibitor-based combination therapy; **(C)** Comparison of PFS between PD-1 inhibitor combined with targeted therapy and PD-1 inhibitor combined with chemotherapy; **(D)** Comparison of OS between PD-1 inhibitor combined with targeted therapy and PD-1 inhibitor combined with chemotherapy.

Among the 37 patients, only 4 patients received PD-1 monotherapy, and the remaining 33 patients received PD-1 immunotherapy-based combination therapy. There was no significant difference in PFS between monotherapy and combination therapy (p=0.13) (**Figure 2A**) and no significant difference in OS between monotherapy and combination therapy (p=0.72) (**Figure 2B**).

### Differences in the efficacy of PD-1 immunotherapy + targeted therapy versus PD-1 immunotherapy + chemotherapy

Of the 37 advanced or unresectable patients, 24 received PD-1 immunotherapy combined with targeted therapy, and 6 received PD-1 immunotherapy combined with AI chemotherapy. There were also 2 patients who received PD-1 immunotherapy + chemotherapy + targeted therapy and 1 patient who received PD-1 immunotherapy + targeted therapy + radiotherapy. These three patients were not included in the analysis of combination therapy due to the small number of cases. Therefore, we compared the efficacy of PD-1 immunotherapy + targeted therapy versus PD-1 immunotherapy + chemotherapy.

The results showed that PFS (p=0.66) (**Figure 2C**) and OS (p=0.96) (**Figure 2D**) were not significantly different between patients receiving PD-1 immunotherapy combined with targeted therapy and patients receiving PD-1 immunotherapy combined with chemotherapy.

### The efficacy of “sensitive” sarcoma and “non-sensitive” sarcoma

**Figure 3:**
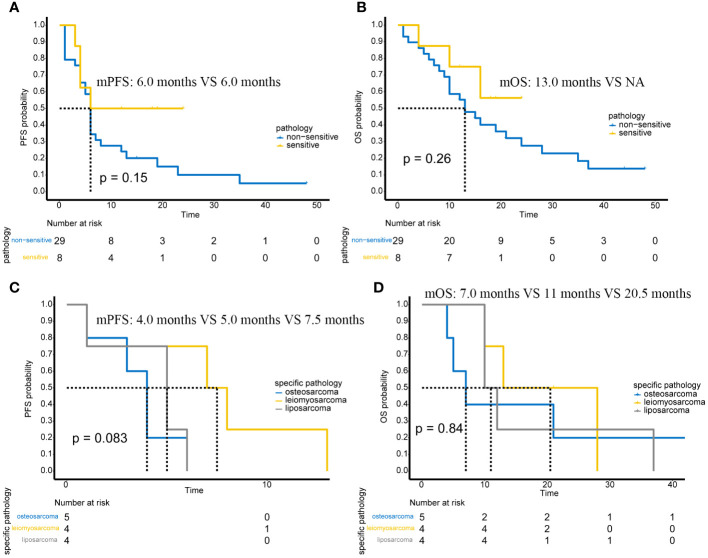
The efficacy of “sensitive” sarcoma and “non-sensitive” sarcoma. **(A)** Comparison of PFS between “Sensitive” and “Non-Sensitive” Sarcomas. **(B)** Comparison of OS between “Sensitive” and “Non-Sensitive” Sarcomas. **(C)** Comparison of PFS in osteosarcoma, leiomyosarcoma, and liposarcoma. **(D)** Comparison of OS in osteosarcoma, leiomyosarcoma, and liposarcoma.

In 37 patients with advanced or unresectable tumors, regarding the pathological subtypes theoretically sensitive to immunotherapy, there were 3 cases of undifferentiated pleomorphic sarcoma, 1 case of undifferentiated sarcoma, 3 cases of alveolar soft part sarcoma, and 1 case of angiosarcoma. Due to the small number of cases, it was not possible to perform survival analysis between subgroups. Therefore, we combined these four subtypes into a ‘sensitive’ sarcoma group, with the other sarcoma subtypes categorized as ‘non-sensitive’ sarcomas. We specifically analyzed whether there was a difference in mPFS and mOS between these two groups of patients (**Figures 3A, B**).

It can be observed that although there was no difference in the mPFS and mOS between ‘sensitive’ and ‘non-sensitive’ sarcomas, there was a trend indicating a better prognosis for ‘sensitive’ sarcomas. With further expansion of the number of cases, positive results might be obtained.

In addition, in the 37 patients with advanced or unresectable tumors, we selected osteosarcoma (5 cases), leiomyosarcoma (4 cases), and liposarcoma (4 cases) for further prognostic analysis due to their higher case numbers in the study. The results show that, possibly due to the small number of patients, there were no significant statistical differences in either mPFS and mOS (**Figures 3 C, D**). However, there was a trend towards poorer PFS in patients with osteosarcoma.

### Efficacy of PD-1 adjuvant therapy

**Figure 4:**
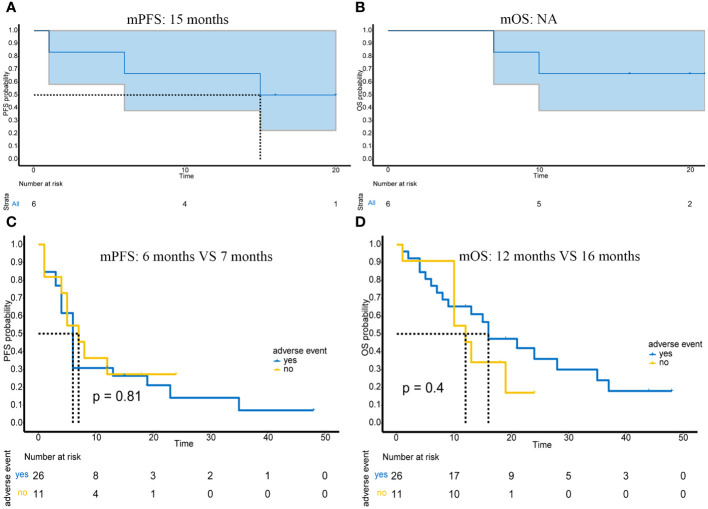
Prognosis of patients receiving postoperative adjuvant immunotherapy and the impact of adverse events on efficacy in advanced or unresectable patients. **(A)** PFS of 6 patients receiving adjuvant therapy; **(B)** OS of 6 patients receiving adjuvant therapy; **(C)** Impact of presence or absence of adverse events on PFS in advanced or unresectable patients; **(D)** Impact of presence or absence of adverse events on OS in advanced or unresectable patients.

Six patients received immunotherapy-based combination therapy after surgery. By the end of follow-up, 3 patients had metastases, and 2 of them died. The mPFS was 15 months (95%CI: 6-NA months) (**Figure 4A**), and the mOS was not reached (**Figure 4B**).

### Adverse events

Of the 43 patients in this study, most adverse events were mild (grade 1-2) and manageable (**Table 2**). Specifically, grade 1 adverse events accounted for 53.4% (31/58), grade 2 adverse events accounted for 29.3% (17/58), grade 3 adverse events accounted for 8.6% (5/58), and grade 4 adverse events accounted for 8.6% (5/58). Myelosuppression was the most common adverse event; it occurred in 24% (14/58) of patients. In addition, other common adverse events included thyroid dysfunction (13.8%, 8/58), capillary hyperplasia (12.1%, 7/58), abnormal liver function (12.1%, 7/58), and cardiac dysfunction (8.6%, 5/58). The most serious grade 4 adverse event was myelosuppression, which occurred in 4 patients and resolved after treatment. In addition, one grade 4 adverse event was hypertension. The presence or absence of adverse reactions also had no impact on mPFS (p=0.81) (**Figure 4C**) or mOS (p=0.4) (**Figure 4D**).

**Table 2:** Adverse events during treatments.

Adverse event	Grade I	Grade II	Grade III	Grade IV	Total
Capillary hyperplasia	7(16.3%)	0	0	0	7(16.3%)
Hand and foot syndrome	2(4.7%)	1(2.3%)	0	0	3(7%)
Rashes and other skin adverse reactions	2(4.7%)	0	0	0	2(4.7%)
Gastrointestinal reactions	3(7.0%)	0	1(2.3%)	0	4(9.3%)
Hypertension	1(2.3%)	1(2.3%)	0	1(2.3%)	3(6.9%)
Immune pneumonia	0	1(2.3%)	0	0	1(2.3%)
Abnormal liver function	5(11.6%)	1(2.3%)	1(2.3%)	0	7(16.2%)
Renal inadequacy	3(7.0%)	1(2.3%)	0	0	4(9.3%)
Myelosuppression	3(7.0%)	5(11.6%)	2(4.7%)	4(9.3%)	14(32.6%)
Cardiac dysfunction	1(2.3%)	4(9.3%)	0	0	5(11.6%)
Thyroid dysfunction	4(9.3%)	3(7.0%)	1(2.3%)	0	8(18.6%)

Some adverse events, such as capillary hyperplasia and hand and foot syndrome, are non-immune-related adverse events, while thyroid dysfunction and pneumonia are immune-related adverse events caused by immunotherapy. Other adverse events, such as cardiac dysfunction, abnormal liver function and renal inadequacy, are difficult to distinguish, as both immunotherapy and chemotherapy or targeted therapy can cause these adverse events

### Some typical cases of PD-1 immunotherapy-based therapy

**Figure 5:**
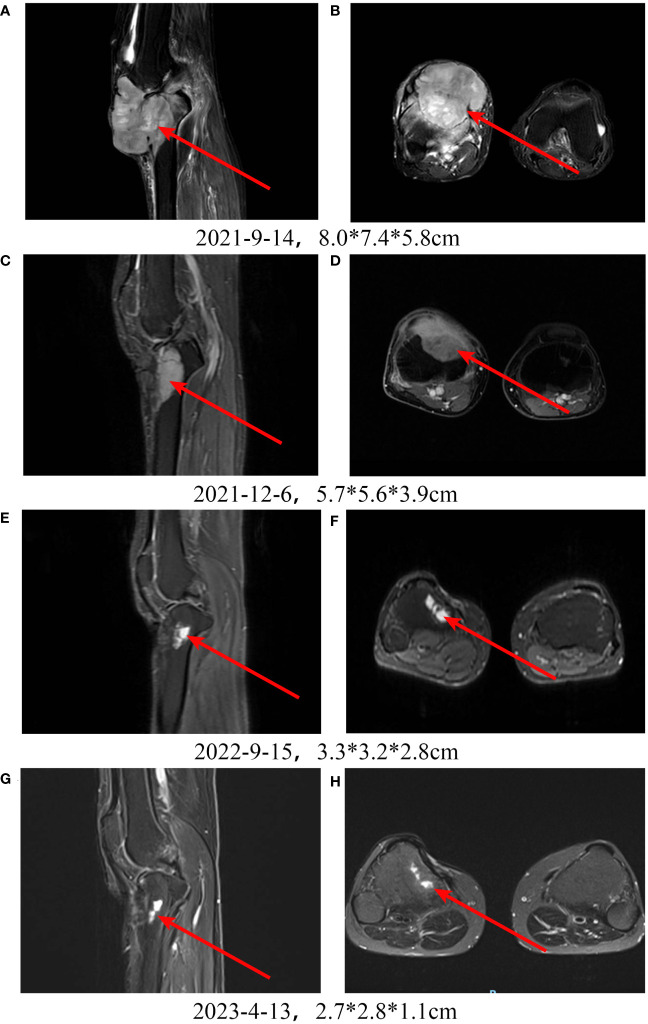
Treatment of a patient with high-grade fibrosarcoma of the right knee with PD-1 inhibitor + chemotherapy + Endostar. **A, B:** Pretreatment MRI scan shows a large tumor in the right knee. **C-H:** After receiving PD-1 inhibitor-based combination therapy, the tumor continuously shrinks.

A patient with high-grade fibrosarcoma of the right knee experienced recurrence 4 months after surgery (**Figures 5A, B**), accompanied by right inguinal lymph node metastasis, and received toripalimab combined with AI and Endostar. During treatment, MRI was regularly reviewed, and the lesions in the right knee continued to shrink. By the end of follow-up, the efficacy was evaluated as PR (**Figures 5C–H**).

**Figure 6:**
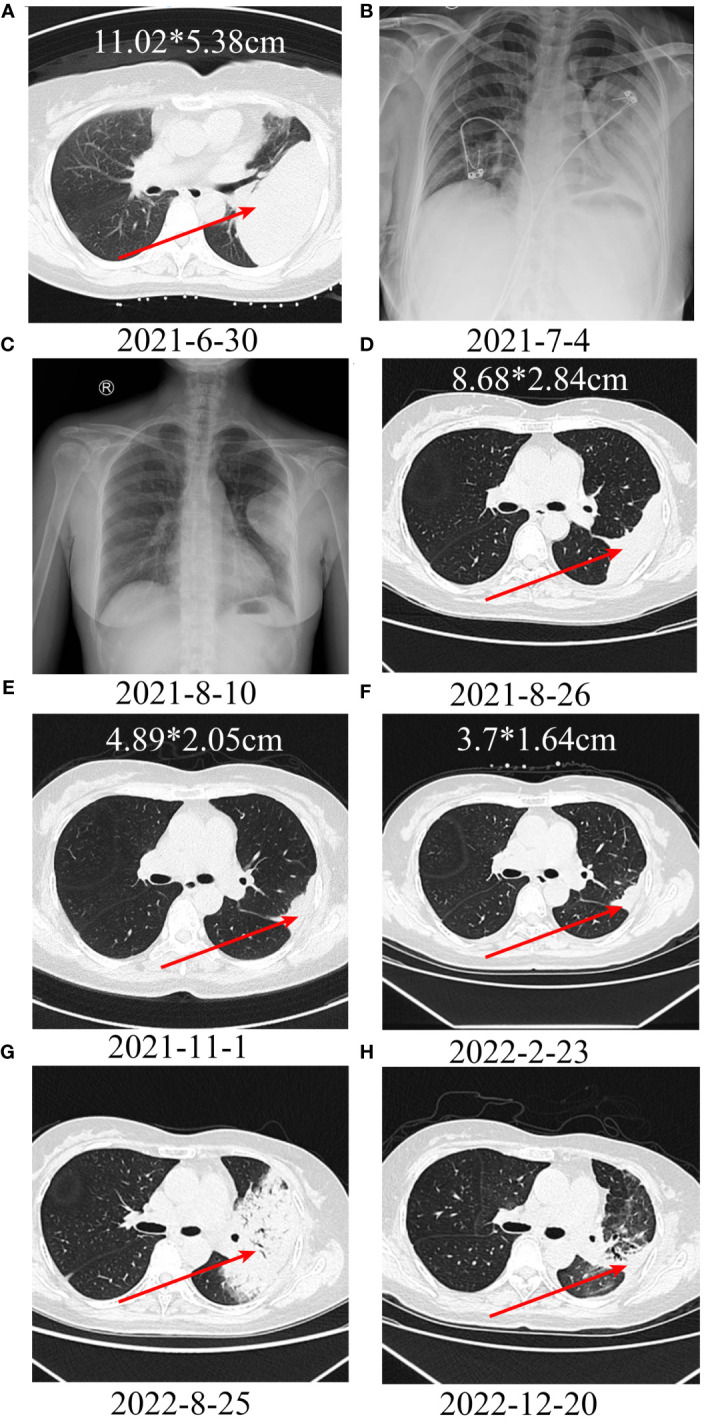
Treatment of a patient with leiomyosarcoma of the left thigh with lung metastases using PD-1 inhibitor + chemotherapy. **A, B:** A large metastatic lesion can be seen in the left lung. **C-F:** After treatment, the metastatic lesion continues to shrink; **(G)** After 1 year of immunotherapy, a large consolidation can be seen in the left lung, suggesting immunotherapy-related pneumonitis. **(H)** After treatment with methylprednisolone and antibiotics, the consolidation resolved.

A patient with a left thigh leiomyosarcoma developed pulmonary metastases with a size of 11.02*5.38 cm after surgery (**Figures 6A, B**). After CT-guided puncture pathology confirmed lung metastasis, albumin-bound paclitaxel + gemcitabine chemotherapy was started, and toripalimab was added from the second cycle. Chest X-ray examination showed a significant reduction in lung metastasis after 1 cycle of toripalimab combined with chemotherapy (**Figures 6C**). Since then, regular CT examination also showed that the tumor continued to shrink (**Figures 6D–F**). After 7 cycles of toripalimab combined chemotherapy, toripalimab monotherapy was initiated. After 1 year of treatment with toripalimab, the CT examination showed a new large consolidation of the left lung, which was considered immune-associated pneumonia and improved after treatment with methylprednisolone and antibiotics (**Figures 6G, H**).

**Figure 7:**
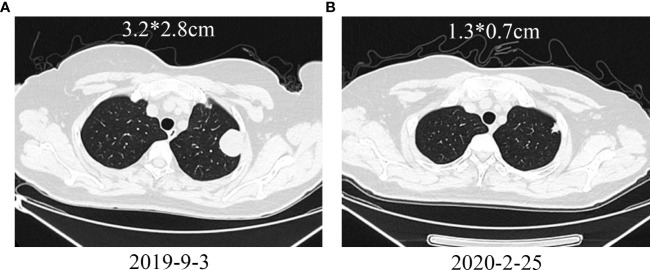
Treatment of a patient with leiomyosarcoma metastasized to the left lung with PD-1 inhibitor. **(A)** Pretreatment shows a metastatic lesion in the left lung; **(B)** After treatment, the lung metastasis shrinks.

Another patient with uterine leiomyosarcoma developed left iliac fossa and left lung metastases after AI chemotherapy. After surgical resection of the left iliac fossa metastasis, GT+ Endostar chemotherapy and stereotactic radiotherapy were performed. Additionally, the left lung metastasis was also enlarged. Considering that the tumor progressed after AI and GT chemotherapy, camrelizumab immunotherapy was started, and chest CT examination after 8 cycles of treatment showed significant shrinkage of lung metastases (**Figures 7A, B**). PD-1 was subsequently discontinued due to the progression of left iliac fossa lesions.

## Discussion

In this two-center immunotherapy-based study, the efficacy and safety of PD-1 inhibitors in patients with sarcoma were evaluated, laying the foundation for a larger exploration of the use of immune checkpoint inhibitors in sarcomas. We divided treatment modalities into palliative therapy for advanced or unresectable patients and adjuvant therapy for postoperative patients, depending on whether the patients underwent surgery. In palliative therapy, not only were overall PFS and OS measured, but the efficacy between different subgroups was also compared, such as PD-L1-negative versus PD-L1-positive patients, osteogenic sarcoma versus soft tissue sarcoma patients, patients with adverse events versus those without adverse events, and patients treated with monotherapy versus those treated with combination therapy. However, among the patients who received postoperative adjuvant therapy, no group comparison was made because the number of cases was too small.

With the increasingly important role of immunotherapy in tumor therapy, immunotherapy for sarcoma has become a hot topic (13). However, there are still some unknowns, such as whether combining multiple immunotherapies or combining immunotherapy with other treatments can improve efficacy. In this study, there was no difference in the efficacy of PD-1 inhibitor monotherapy and combination therapy based on PD-1 inhibitors, and there was no difference in the efficacy of PD-1 inhibitors combined with targeted therapy and PD-1 inhibitors combined with chemotherapy.

A retrospective study pooled 9 studies of PD-1/PD-L1 inhibitors containing 384 soft tissue sarcoma patients, of whom 153 (39.8%) received either a PD1 or PD-L1 inhibitor as a single agent. The overall ORR was 15.1%, the ORR in PD-1/PD-L1 monotherapy patients was 18.7%, and the ORR in combination therapy patients was 13.4%, suggesting that combination therapy had no significant effect on improving ORR (14). In the clinical trial Alliance A091401, which also explored the efficacy of the combination of two immune checkpoint inhibitors, nivolumab and ipilimumab, the results showed that 6 of 38 combination-treated patients had a response with a mOS of 14.3 months, while 2 of 38 patients treated with nivolumab monotherapy had a response with a mOS of 10.7 months (15). In addition, several studies have explored the efficacy of immunotherapy combined with targeted therapy. One clinical trial evaluated the efficacy of pembrolizumab in combination with axitinib in patients with advanced sarcoma, with a mPFS of 4.7 months and a mOS of 18.7 months, but did not compare the efficacy of combination therapy with pembrolizumab monotherapy (9). Another study evaluated the efficacy of nivolumab combined with sunitinib in the treatment of advanced soft tissue sarcoma and enrolled 68 patients. Among 58 patients who were eligible for analysis, the ORR was 21%, and the 6-month progression-free survival was 48%, but no patients treated with nivolumab alone were included (16, 17). Another study evaluated the efficacy of camrelizumab combined with apatinib in patients with advanced osteosarcoma and enrolled 43 patients. At the end of follow-up, the 6-month PFS rate was 50.9%, and the objective response rate was 20.9% (9/43). Again, no comparison was made with camrelizumab monotherapy (18). Therefore, more clinical trials are needed to compare the efficacy of immunotherapy combined with other treatments and immunotherapy alone. In this study, a total of 43 patients who received immunotherapy were included, of which 37 were patients with advanced or unresectable sarcomas. Among these 37 patients, only 4 received PD-1 inhibitors monotherapy, while the rest were treated with combination therapies. This may be because immunotherapy is still not the first-line treatment for patients with advanced sarcoma, often requiring the use of additional drugs in combination. Perhaps due to the significant difference in the number of cases, there was no clear difference in the prognosis between patients receiving monotherapy and those receiving combination therapy. We will conduct further analysis with more patients receiving immunotherapy in the future to obtain more accurate conclusions.

In terms of immunotherapy-sensitive subtypes, the response rate of undifferentiated pleomorphic sarcoma, alveolar soft part sarcoma and liposarcoma was relatively high, while the response rate of osteosarcoma and leiomyosarcoma was low (14). The results of our study showed that there was no significant difference in the efficacy of osteogenic sarcoma and soft tissue sarcoma. However, in the case of specific soft tissue sarcoma subtypes, the number of cases was too small, such as only 3 cases of undifferentiated pleomorphic sarcoma and 4 cases of liposarcoma, so the difference in efficacy between specific pathological subtypes could not be assessed. In future clinical work, we will carry out the corresponding evaluation after collecting enough cases.

In addition, there was no significant difference in efficacy between PD-L1-negative and PD-L1-positive patients in our study. In fact, when focusing on the relationship between PD-L1 expression and prognosis, some studies have shown that high PD-L1 expression is associated with a worse prognosis, while other studies have shown the opposite, such as a meta-analysis of, 1451 patients showing that high PD-L1 expression is associated with poorer overall survival (p < 0.0001) and poorer EFS (p<0.0001) (17, 19). Additionally, the correlation between PD-L1 expression and immunotherapy efficacy in the treatment of immune checkpoint inhibitors in sarcoma is unclear. In a report that pooled several studies, 154 patients were tested for PD-L1 expression, of which 21 patients were positive, 20 were evaluable for efficacy, 6 were effective, and the ORR was 30%, while of the remaining 133 patients who were negative for PD-L1, only 9 were effective, and the ORR was 6.77% (14). In a clinical trial of camrelizumab combined with apatinib for the treatment of osteosarcoma, the results also demonstrated that patients with a PD-L1 score >5% had a longer progression-free survival (PFS) (p=0.004) (18). However, PD-L1 expression does not seem to be necessary for treatment efficacy. In another clinical trial for advanced osteosarcoma, the only patient who achieved a partial response (PR) had negative PD-L1 expression (20). In addition, the prognostic value of PD-L1 expression may also be related to pathological subtypes. For example, PD-L1 expression shows no significant correlation with prognosis in leiomyosarcoma and retroperitoneal liposarcoma but is associated with survival in alveolar rhabdomyosarcoma (1). Therefore, PD-1/PD-L1 as a biomarker for immunotherapy has limitations.

Other studies have explored the impact of biomarkers other than PD-L1 expression on the efficacy of immunotherapy in sarcomas. A retrospective study that included 135 patients found that among those receiving monotherapy with immunotherapy, patients with a change in the neutrophil-to-lymphocyte ratio (NLR) of less than 5 had improved overall survival (OS) (p=0.002), while this phenomenon was not observed in combination immunotherapy; patients experiencing adverse events from immunotherapy, especially colitis (p=0.009), hepatitis (p=0.048), or dermatitis (p=0.003), had improved PFS (21). Gene Expression Signatures (GES) are a set of specific genes that have been used in various other cancers to predict prognosis and treatment outcomes. In soft tissue sarcomas, Petitprez et al. identified a signature associated with B-cell immunity features, indicating a high immune infiltration status in patients, which correlates with better efficacy of pembrolizumab treatment (22). Indoleamine 2,3-Dioxygenase (IDO) is a heme-containing enzyme that has been proven to be associated with the suppression of T cell function and the upregulation of regulatory T cell activity (23, 24). In sarcomas, studies have shown that IDO is related to PFS and OS, but its role as a biomarker for predicting the efficacy of immunotherapy still requires further investigation (25). Additionally, tertiary lymphoid structures also have the potential to serve as biomarkers for predicting the efficacy of immunotherapy in sarcomas (26).

This study has several limitations. First, there is more than one type of drug for immune checkpoint inhibitors, chemotherapy, and targeted therapy. For example, the immune checkpoint inhibitors used include nivolumab, camrelizumab, and toripalimab, while chemotherapy regimens include albumin-bound paclitaxel plus gemcitabine and ifosfamide plus doxorubicin, and targeted drugs include pazopanib, apatinib, and anlotinib. This diversity in treatment options may introduce biases in evaluating the effectiveness of immune checkpoint inhibitor-based combination therapy. Second, the number of included patients was limited, and there was a variety of pathological types. Due to the rarity of sarcomas, it is difficult to collect a sufficient number of cases, and conducting prospective clinical trials or retrospective studies specifically targeting a single subtype of sarcoma is even more challenging. This is a common limitation in sarcoma-related research. Third, regarding the biomarkers for immune checkpoint inhibitors, due to reasons related to specimen collection, we only evaluated the expression of PD-1 in 13 patients, among whom 3 were positive and 10 were negative. The results also showed no association between PD-1 expression and treatment efficacy. The limited number of cases may lead to biased results. Furthermore, we did not evaluate the relationship between tumor mutation burden (TMB)/tumor neoantigen burden (TNB), microsatellite instability (MSI), and immunotherapy. In future studies, when we collect a sufficient number of cases and a sufficient amount of data, we will reanalyze these aspects.

In conclusion, in this two-center study, we systematically evaluated the efficacy and safety of immune checkpoint inhibitor-based combination therapy in sarcoma patients. The results demonstrated that immunotherapy can improve patient prognosis while being well tolerated in terms of adverse events, further confirming the effectiveness of immunotherapy. Additionally, it is necessary to recruit more patients to identify the pathological subtypes that respond effectively to immunotherapy to facilitate more targeted treatments.

## Data availability statement

The raw data supporting the conclusions of this article will be made available by the authors, without undue reservation.

## Ethics statement

The studies involving humans were approved by Tianjin Medical University Cancer Institute and Hospital. The studies were conducted in accordance with the local legislation and institutional requirements. The participants provided their written informed consent to participate in this study.

## Author contributions

ZL: Formal Analysis, Investigation, Methodology, Writing – original draft. JT: Formal Analysis, Writing – original draft. TL: Formal Analysis, Writing – original draft. HL: Formal Analysis, Writing – original draft. TL: Writing – review and editing. CZ: Writing – review and editing. RX: Writing – review and editing. ST: Writing – review and editing. YY: Writing – review and editing. JZ: Writing – review and editing. WX: Writing – review and editing. GZ: Writing – review and editing. JL: Writing – review and editing. WY: Writing – review and editing. JY: Writing – review and editing.

## Supplementary material

Supplementary Figure 1 Expression of PD-L1. A: High expression of PD-L1; B: Low expression of PD-L1.
