# Supplementary figures and images for: Evaluation of the efficacy and safety of immunotherapy in sarcoma: a two-center study
Source: Front Immunol. 2024 Mar 22;15:1292325. doi: 10.3389/fimmu.2024.1292325 (PMC10995229; doi:10.3389/fimmu.2024.1292325)

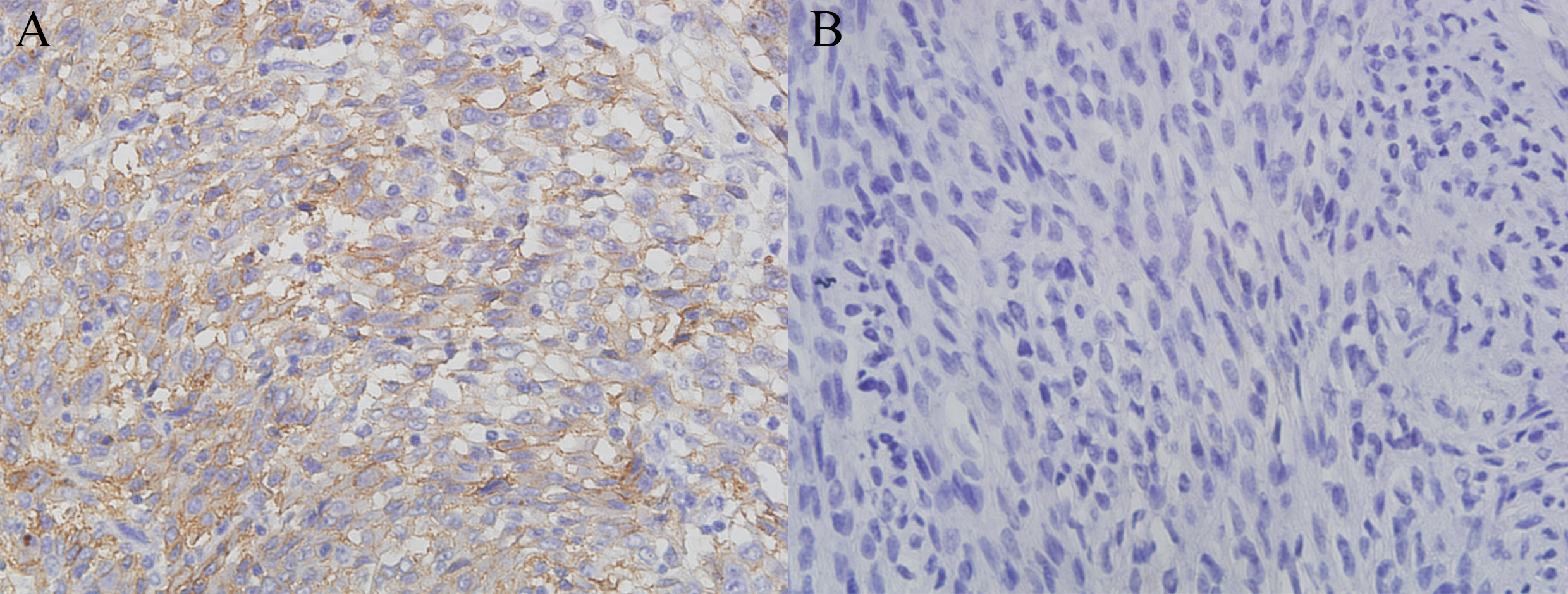

Supplement: Supplementary Figure 1 — Expression of PD-L1. A: High expression of PD-L1; B: Low expression of PD-L1. [file Image_1.tif]
