# Supplementary material for: Evaluation of the efficacy and safety of immunotherapy in sarcoma: a two-center study
Source: Front Immunol. 2024 Mar 22;15:1292325. doi: 10.3389/fimmu.2024.1292325 (PMC10995229; doi:10.3389/fimmu.2024.1292325)
Supplement: Supplementary file 2 [file Table_1.docx]

**Supplementary Table 1** The efficacy of 37 patients with advanced or unresectable tumors

| Patients | Subtype | Efficacy at 12th week | Final efficacy |
| --- | --- | --- | --- |
| Patient 1 | malignant solitary fibrous tumor | SD | PD |
| Patient 2 | malignant peripheral nerve sheath tumor | SD | PD |
| Patient 3 | malignant peripheral nerve sheath tumor | SD | PD |
| Patient 4 | osteosarcoma | PR | PD |
| Patient 5 | osteosarcoma | SD | PD |
| Patient 6 | osteosarcoma | SD | PD |
| Patient 7 | osteosarcoma | PD | PD |
| Patient 8 | osteosarcoma | PD | PD |
| Patient 9 | rhabdomyosarcoma | PD | PD |
| Patient 10 | rhabdomyosarcoma | PD | PD |
| Patient 11 | rhabdomyosarcoma | PD | PD |
| Patient 12 | synovial sarcoma | SD | PD |
| Patient 13 | synovial sarcoma | SD | PD |
| Patient 14 | synovial sarcoma | SD | PD |
| Patient 15 | leiomyosarcoma | PR | PD |
| Patient 16 | leiomyosarcoma | SD | PD |
| Patient 17 | leiomyosarcoma | SD | PD |
| Patient 18 | leiomyosarcoma | PR | PD |
| Patient 19 | chondrosarcoma | SD | PD |
| Patient 20 | chondrosarcoma | PR | PD |
| Patient 21 | epithelioid sarcoma | CR | CR |
| Patient 22 | epithelioid sarcoma | SD | CR |
| Patient 23 | clear cell sarcoma | PD | PD |
| Patient 24 | clear cell sarcoma | PD | PD |
| Patient 25 | undifferentiated pleomorphic sarcoma | PD | PD |
| Patient 26 | undifferentiated pleomorphic sarcoma | PD | PD |
| Patient 27 | undifferentiated pleomorphic sarcoma | SD | PD |
| Patient 28 | undifferentiated sarcoma | SD | SD |
| Patient 29 | fibrosarcoma | PR | PR |
| Patient 30 | alveolar soft part sarcoma | SD | CR |
| Patient 31 | alveolar soft part sarcoma | SD | SD |
| Patient 32 | alveolar soft part sarcoma | SD | SD |
| Patient 33 | angiosarcoma | SD | PD |
| Patient 34 | liposarcoma | SD | PD |
| Patient 35 | liposarcoma | SD | PD |
| Patient 36 | liposarcoma | PD | PD |
| Patient 37 | liposarcoma | SD | PD |
